# Study of scattered radiation during fluoroscopy in hip surgery*

**DOI:** 10.1590/0100-3984.2014.0146

**Published:** 2016

**Authors:** Oksana Lesyuk, Patrick Emmanuel Sousa, Sónia Isabel do Espirito Santo Rodrigues, António Fernando Abrantes, Rui Pedro Pereira de Almeida, João Pedro Pinheiro, Kevin Barros Azevedo, Luís Pedro Vieira Ribeiro

**Affiliations:** 1 Radiology Technician, Professor in the Department of Medical Imaging and Radiotherapy of the Escola Superior de Saúde da Universidade do Algarve (ESSUAlg), Faro, Portugal; 2 PhD, Physics Engineer, Professor in the Department of Medical Imaging and Radiotherapy of the Escola Superior de Saúde da Universidade do Algarve (ESSUAlg), Faro, Portugal; 3 MSc, Radiology Technician, Professor in the Department of Medical Imaging and Radiotherapy of the Escola Superior de Saúde da Universidade do Algarve (ESSUAlg), Faro, Portugal; 4 PhD, Professor and Head of the Department of Medical Imaging and Radiotherapy of the Escola Superior de Saúde da Universidade do Algarve (ESSUAlg), Faro, Portugal; 5 MsC, Radiology Technician, Graduate Student at the Universidad de Murcia, Murcia, Spain, Professor in the Department of Medical Imaging and Radiotherapy of the Escola Superior de Saúde da Universidade do Algarve (ESSUAlg), Faro, Portugal; 6 MSc, Doctoral Student at the Universidade de Coimbra, Coimbra, Portugal, Professor in the Department of Medical Imaging and Radiotherapy of the Escola Superior de Saúde da Universidade do Algarve (ESSUAlg), Faro, Portugal; 7 PhD, Professor in the Department of Medical Imaging and Radiotherapy of the Escola Superior de Saúde da Universidade do Algarve (ESSUAlg), Faro, Portugal

**Keywords:** Radiation, ionizing, Operating rooms, Scattering, radiation, Radiation protection, Radiology, interventional

## Abstract

**Objective:**

To measure the scattered radiation dose at different positions simulating hip surgery.

**Materials and Methods:**

We simulated fluoroscopy-assisted hip surgery in order to study the distribution of scattered radiation in the operating room. To simulate the patient, we used a anthropomorphic whole-body phantom, and we used an X-ray-specific detector to quantify the radiation. Radiographs were obtained with a mobile C-arm X-ray system in continuous scan mode, with the tube at 0º (configuration 1) or 90º (configuration 2). The operating parameters employed (voltage, current, and exposure time) were determined by a statistical analysis based on the observation of orthopedic surgical procedures involving the hip.

**Results:**

For all measurements, higher exposures were observed in configuration 2. In the measurements obtained as a function of height, the maximum dose rates observed were 1.167 (± 0.023) µSv/s and 2.278 (± 0.023) µSv/s in configurations 1 and 2, respectively, corresponding to the chest level of health care professionals within the operating room. Proximal to the patient, the maximum values were recorded in the position occupied by the surgeon.

**Conclusion:**

We can conclude that, in the scenario under study, health care professionals workers are exposed to low levels of radiation, and that those levels can be reduced through the use of personal protective equipment.

## Abstract (pt)

**Objetivo:**

Medir a intensidade da dose de radiação espalhada em diferentes posições simulando uma intervenção cirúrgica no quadril.

**Materiais e Métodos:**

Simulou-se uma intervenção cirúrgica no quadril com apoio da fluoroscopia para estudar a distribuição da radiação espalhada no bloco operatório. Para simular o paciente foi utilizado um simulador antropomórfico de corpo inteiro e para medir a radiação utilizou-se um detector específico para medir raios X. Realizaram-se incidências com um equipamento de raios X tipo arco em C móvel, em modo de escopia contínua, com a ampola a 0º (configuração 1) e a 90º (configuração 2). Os parâmetros operacionais utilizados (voltagem, corrente, tempo de exposição) foram determinados por meio de um estudo estatístico resultante da observação de cirurgias ortopédicas de quadril.

**Resultados:**

Em todas as medições observaram-se exposições mais elevadas na configuração 2. Nas medições em função da altura, observaram-se os valores máximos da taxa de dose de 1,167 (± 0,023) µSv/s e 2,278 (± 0,023) µSv/s nas configurações 1 e 2, respectivamente, correspondendo à altura do tórax dos profissionais. No estudo em torno do paciente os valores máximos registraramse na posição ocupada pelo médico cirurgião.

**Conclusão:**

Concluiu-se que a exposição à radiação dos profissionais é baixa, podendo ainda ser reduzida mediante o uso de equipamentos de proteção individual.

## INTRODUCTION

The use of ionizing radiation for diagnostic and treatment purposes has increased due to the development of new equipment and easier access to radiologic exams^(1)^. Medical activities such as interventional radiology involve exposing patients and health care professionals to radiation, and radiation protection is therefore necessary in order to reduce the levels of that exposure.

The involvement of professionals from various areas, without specific training in the field of radiation protection, can lead to excessive exposure to ionizing radiation in the operating room^(2,3)^. Previous studies have indicated that nonradiologist physicians possess heterogeneous, inadequate knowledge of ionizing radiation, suggesting that there is room for improvement^(4)^.

Ionizing radiation produces lesions in cells and can have deterministic or stochastic effects^(5,6)^. To minimize radiation exposure, there are laws stipulating dose limits for workers who are exposed while exercising their professions. The average annual effective dose received by a worker should not exceed 20 mSv (100 mSv in a period of five years) and may not surpass 50 mSv in any given year. The annual equivalent dose should not exceed 500 mSv for the skin and extremities and 15 mSv for the lens of the eye. According to Portuguese law^(7)^, effective doses above 1.5 mSv/month should be investigated.

There are many limitations that make proper dose monitoring difficult. Such limitations include failure to use personal dosimeters and the incorrect use of such dosimeters, as well as their inherent limitations, such as detecting radiation at a single angle, which depends on the position of the device in relation to the source of the radiation^(5)^.

Exposure to radiation has been given attention at general radiology centers. However, work conditions involving ionizing radiation exposure are not routinely monitored during diagnostic or therapeutic orthopedic interventions^(5)^.

According to information published on the International Atomic Energy Agency website^(8)^, there have been numerous studies investigating the levels of ionizing radiation received by medical professionals during procedures that carry a high risk of such exposure, including those related to hemodynamics, angiography, or gastroenterology. However, there is still a need for studies of other, low-risk, procedures, such as orthopedic interventions, specifically those involving the backbone and hip, where there is greater exposure to ionizing radiation^(9)^.

It is pertinent to study the distribution of scattered radiation in the operating room during a simulated fluoroscopy-guided orthopedic intervention, to evaluate the intensity of the scattered radiation in different zones of the operating room, and to identify factors which influence professionals' exposure during interventions, thus establishing radiation protection recommendations to apply the "as low as reasonably achievable" principles with greater efficiency.

## MATERIALS AND METHODS

Between January 1 and April 30 of 2014, a study of interventional radiology procedures in orthopedics was conducted at the Faro Branch of the Algarve Hospital Center, in the city of Faro, Portugal. We evaluated the respective operating parameters (voltage, current, and fluoroscopy time) of the dose-area product received by the patient, the data related to the positions occupied by the professionals, and the configuration of the C-arm around the table, in order to determine which procedure produces the most radiation and to evaluate the image acquisition conditions.

After the statistical study described above, the scattered radiation dose rate was measured as a function of height, distance, and the angle between the simulator and the detector in configuration 1 (tube at 0º) and configuration 2 (tube at 90º), during a simulation of fluoroscopy-guided hip surgery. An AR10A whole-body phantom (Adam,Rouilly Limited, Kent, England) was used as a surrogate for the patient^(10)^.

We employed a radiation monitor AT1123 (Atomtex; Minsk, Belarus). The monitor was used in order to measure the background dose rate, referred to throughout the text as the dose rate, with a maximum intrinsic uncertainty of ± 15%, in continuous mode^(11)^. The fluoroscopy equipment used in the study was a Philips model BV300 (Philips Medical Systems; Best, the Netherlands), with the voltage set at 80 kV and quality controlled, the maximum deviation being ± 0.6%, which is well within the ± 10% tolerance defined by law^(12)^.

**Figure 1:**
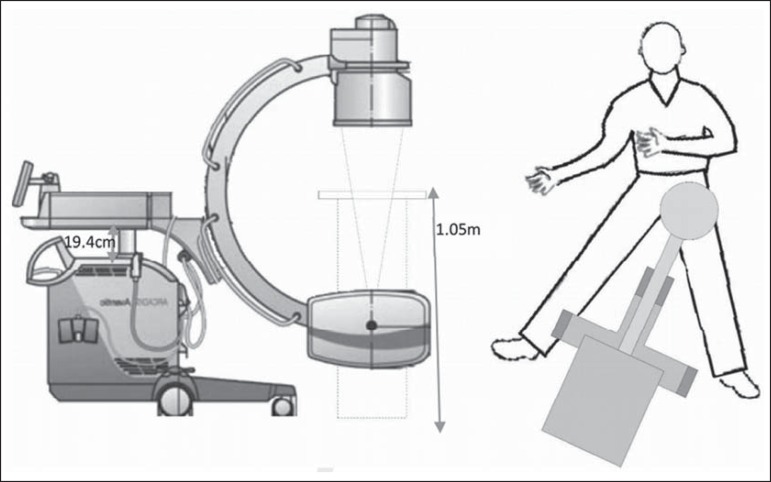
Schematic illustration of the positioning of the phantom and the C-arm fluoroscopy equipment.

The phantom was positioned to simulate a surgical procedure involving the left hip, with the lower left member extended and lower right member in maximum abduction. The table was placed at a height of 1.05 m above the operating room floor, and the fluoroscopy equipment was placed with its longitudinal axis parallel to the longest axis of the lower right member, centered over the left hip joint (Figure 1).

The operating parameters for voltage and current were in accordance with the results of the statistical study, in two configurations of the C-arm: 67 kV and 2.4 mA, respectively, with the tube at 0º (configuration 1), and 76 kV and 2.8 mA, respectively, with the tube at 90º (configuration 2). The radiation reading was registered after the radiation beam had stabilized, typically after it had been on for 5 s.

### Variation in the dose rate as a function of height

For the study of the scattered radiation dose rate as a function of the dosimeter height, the initial settings for the table, equipment, and phantom were maintained, and the detector was placed at a fixed distance of 25 cm from the center of the exposure field, the approximate position of the lead surgeon. Readings were taken for both configurations, at a 90º angle to the median sagittal line of the phantom, dose readings being taken between 0.10 m and 1.80 m, changing the position of the detector in increments of 10 cm.

### Variation in the dose rate as a function of distance

For the study of the scattered radiation dose rate as a function of distance, the equipment and the phantom were maintained in their original positions, and the radiation monitor was placed at a fixed height of 1.25 m, corresponding to the plane of incidence of the radiation beam on the phantom for configuration 1, at a 90º angle to the median sagittal line, only the distance between the phantom and the detector varying in both configurations. The doses were measured between 0.25 m and 1.65 m, the detector being repositioned in increments of 10 cm.

### Variation in the dose rate around the phantom

For the study of the scattered radiation readings around the phantom, the initial positioning was maintained, and the radiation monitor was placed at 1.0 m from the center of the exposure field, at a height of 1.25 m in the plane of incidence of the radiation beam in configuration 1. The position of the detector was changed in 15º increments, the 0º angle corresponding to the median sagittal line in the direction of the head.

## RESULTS

The statistical study conducted prior to the dose rate readings involved a sample of 55 orthopedic interventions and showed that the procedure that emits the most scattered radiation is hip surgery, because it is the most common intervention and produces the highest dose values. The mean voltage and current were 67 kV and 2.4 mA, respectively, for configuration 1, compared with 76 kV and 2.8 mA, respectively, for configuration 2. The mean fluoroscopy time per intervention was 27 s.

### Variation in the dose rate as a function of height above the operating room floor

**Figure 2:**
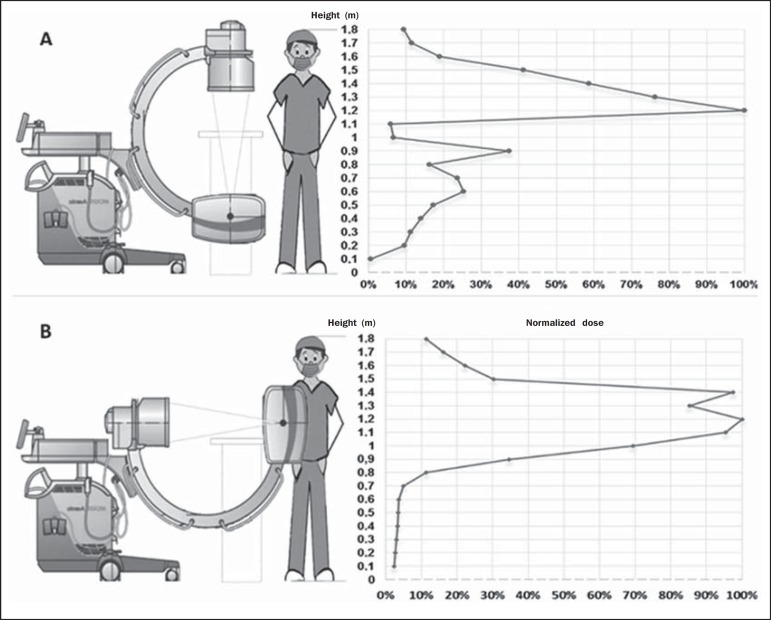
Graphic illustrations of the variation in the dose rate as a function of height, with the tube at 0º (**A**) and at 90º (**B**) at a distance of 25 cm from the center of the exposure field, at a 90° angle to the median sagittal line of the phantom.

The scattered radiation dose rate readings as a function of height are shown for configurations 1 and 2 in Figures 2A and 2B, respectively. At chest level, the maximum dose rates were 1.167 ± 0.023 µSv/s and 2.278 ± 0.023 µSv/s in configurations 1 and 2, respectively. At the thyroid level, the mean dose rates registered were 0.481 ± 0.010 µSv/s and 0.692 ± 0.007 µSv/s in configurations 1 and 2, respectively, compared with 0.133 ± 0.0013 µSv/s and 0.367 ± 0.011 µSv/s, respectively, at the level of the lens of the eye.

### Variation in the dose rate as a function of distance

**Figure 3:**
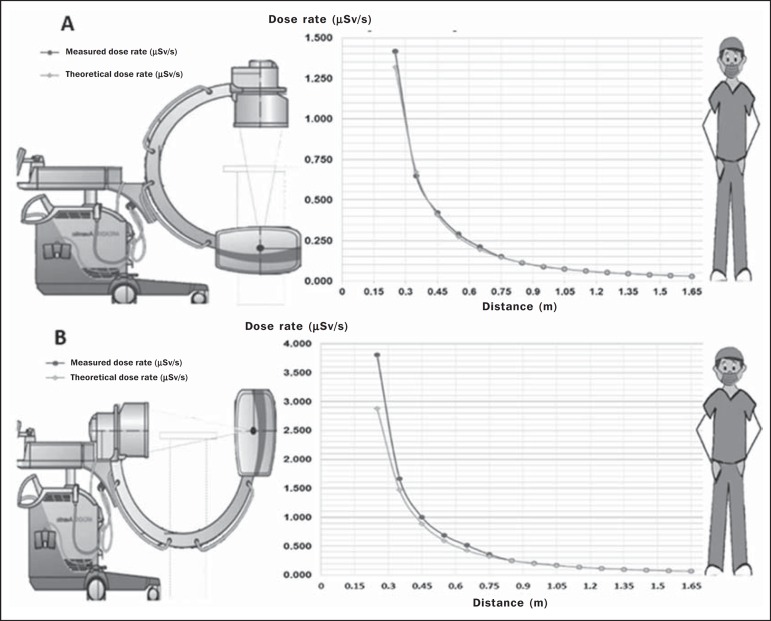
Graphic illustrations of the variation in the dose rate as a function of distance, at a height of 1.25 m and at a 90º angle to the median sagittal line of the phantom, for configuration 1 (**A**) and configuration 2 (**B**).

For configurations 1 and 2, the scattered radiation dose rate readings as a function of the distance from the center of the exposure field are shown in Figures 3A and 3B, respectively. We also compared the experimental and theoretical distance values obtained by the inverse square law.

According to the general rule of irradiance, an extended source may be considered a point source if the distance from the source is greater than five times its diameter^(9)^. Therefore, to calculate the theoretical values, the value measured at the greatest distance was used, allowing the application of the inverse square law.

We observed differences between the measured values and the theoretical values, those differences being more pronounced in configuration 2 and for distances less than 1.0 m.

### Variation in the dose rate around the phantom

**Figure 4:**
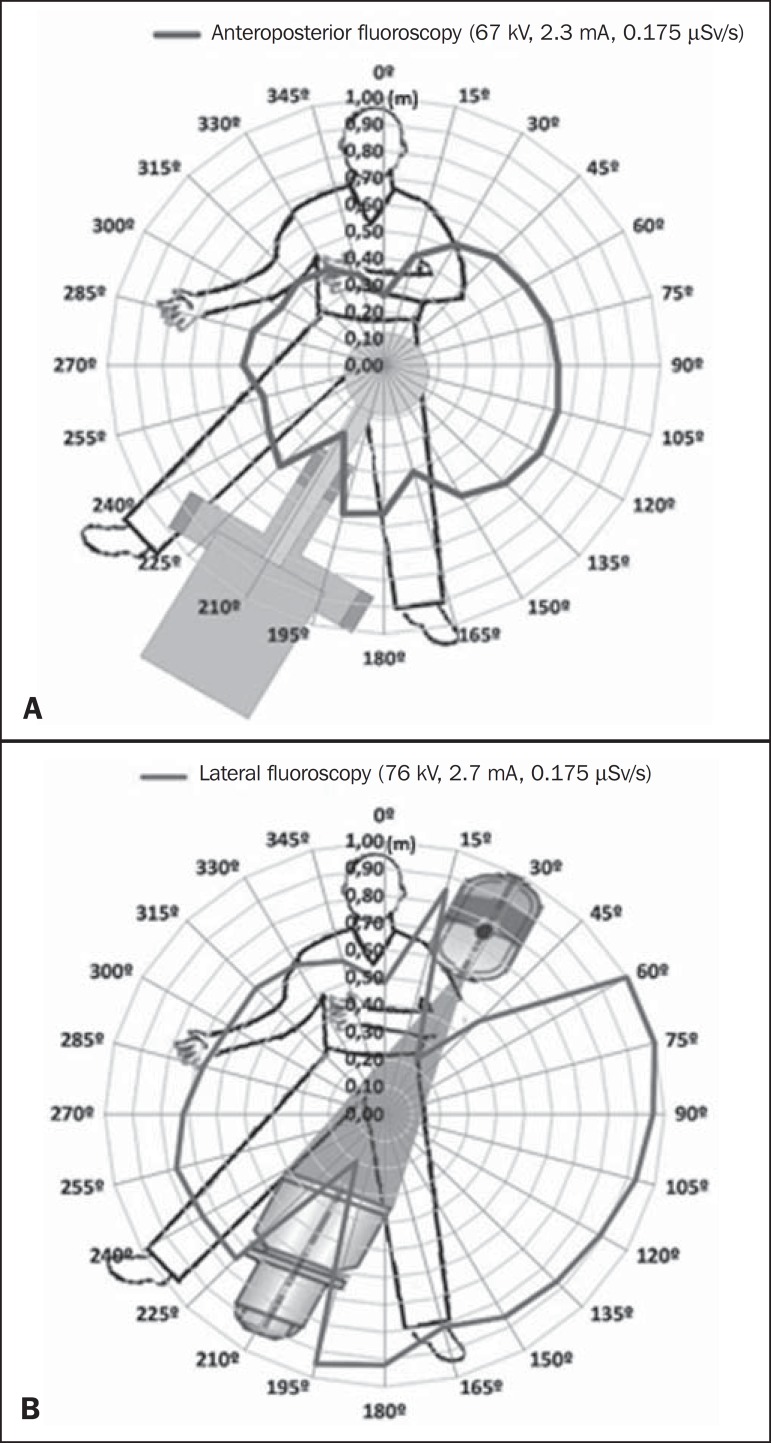
Isodose curve around the phantom traced for configuration 1 (**A**) and configuration 2 (**B**).

Considering the scattered radiation dose rate readings around the phantom (Table 1), we used the inverse square law formula to estimate, for each angle, the distance at which the detector should be to receive the maximum scattered radiation dose rate registered (0.175 µSv/s), thus tracing the isodose curves for configurations 1 and 2, as shown in Figures 4A and 4B, respectively.

**Table 1:** Rates of scattered radiation doses around the phantom.

	Configuration 1 (0º)			Configuration 2 (90º)	
	Dose rate	Distance		Dose rate	Distance
Position	(µSv/s)	(m)		(µSv/s)	(m)
0º	0.012	0.26		0.041	0.48
15º	0.031	0.42		0.128	0.85
30º	0.048	0.52		0.009	0.23
45º	0.057	0.57		0.041	0.48
60º	0.062	0.59		0.175	1.00
75º	0.067	0.62		0.175	1.00
90º	0.070	0.63		0.161	0.96
105º	0.074	0.65		0.150	0.93
120º	0.073	0.65		0.139	0.89
135º	0.068	0.62		0.133	0.87
150º	0.054	0.56		0.131	0.86
165º	0.029	0.41		0.114	0.81
180º	0.053	0.55		0.147	0.92
195º	0.056	0.57		0.158	0.95
210º	0.015	0.29		0.007	0.20
225º	0.050	0.53		0.097	0.75
240º	0.040	0.48		0.097	0.75
255º	0.036	0.45		0.103	0.77
270º	0.045	0.51		0.092	0.72
285º	0.043	0.49		0.079	0.67
300º	0.033	0.43		0.071	0.64
315º	0.034	0.44		0.073	0.65
330º	0.029	0.41		0.069	0.63
345º	0.021	0.35		0.059	0.58

Figure 4 shows an anisotropic dose distribution around the phantom, indicated by the line that connects the points of equal doses at different distances. In both configurations, the highest doses registered were to the left of the patient and the maximum dose rate was 0.175 µSv/s, registered for the incidence in profile, at a distance of 1.0 m, at 60º and 75º.

### Estimate of the effective dose received by professionals

On the basis of the dose rates measured as a function of the angle and of the distance at which where professionals were from the center of exposure, we estimated the effective dose received at the position of each professional, assuming that the members of the team maintain the same positions throughout the surgical procedure.

In calculating the effective doses, we assumed that the overall duration of an intervention was 27 s. The interventions evaluated were distributed equally between configurations 1 and 2 (Table 2).

**Table 2:** Estimate of the effective dose received by health professionals based on the dose rate measured at 1.25 m in relation to the patient plane.

	Distance	Dose rate in configuration 1	Dose rate in configuration 2	Dose per intervention
Professional	(m)	(µSv/s)	(µSv/s)	(µSv)
Lead surgeon	0.3	0.775	1.790	34.6
Attending physician	0.5	0.293	0.556	11.5
Instrument nurse	1.6	0.029	0.059	1.2
Nurse anesthetist	1.2	0.015	0.041	0.8
Anesthesiologist	1.2	0.015	0.041	0.8
Circulating nurse	2.4	0.009	0.026	0.5
Radiology technician	1.9	0.004	0.002	0.8

On the basis of previous studies, it is estimated that approximately 282 surgical interventions involving the hip are performed per year in the Orthopedics Department of the Faro Branch of the Algarve Hospital Center. Assuming that there are five surgical teams performing these interventions, each team therefore carrying out approximately 57 fluoroscopy-guided hip interventions procedures per year, we estimated that the lead surgeon receives a cumulative annual scattered radiation dose of 1.974 mSv, compared with 0.653 mSv for the attending physician.

## DISCUSSION

Fluoroscopy is frequently used by medical professionals. Therefore, it is necessary to raise awareness in relation to the risks of ionizing radiation, as well as to encourage the use of personal protective equipment and greater attention to radiation protection recommendations in order to reduce the doses received during medical procedures^(1)^.

A previous study carried out by our group indicated that the medical field in which fluoroscopy is most frequently requested is orthopedics, primarily the subspecialty of hip surgery. Therefore, we decided to study the distribution of scattered radiation during those procedures and estimate the effective doses of radiation received by the different professionals involved.

In relation to the parameters used in this study during the radiation beam simulation and exposure time, the mean exposure time observed in the present study was similar to the 26 s reported by Alonso et al.^(13)^. In addition, our values for current and voltage were similar to those reported by Fuchs et al.^(14)^.

The readings for the dose rate as a function of height in relation to the floor of the operating room showed that the radiation intensity was greatest at the level of the chest of the lead surgeon. That was true for both configurations.

Assuming that the exposure duration at the level of the lens of the eye is 30 s, we estimated that the equivalent dose to the eyes is 7.5 µSv per intervention, which is below the 11.2-45.5 µSv range of values indicated in the study conducted by Fuchs et al.^(14)^. It should be borne in mind that the annual equivalent dose for the lens of the eye is 15 mSv per year^(15)^.

At the thyroid level, the estimated dose was 17.58 µSv per intervention under the same conditions described above. That is within the 16.7-67.9 µSv dose range indicated in the study conducted by Fuchs et al.^(14)^.

For the dose rate as a function of distance, there was a difference between the experimental and theoretical values for short distances from the exposure field. Therefore, the inverse square law underestimates the true dose rate in that simulation.

In relation to the dose rate around the phantom indicated by the isodose curves, we observed a 210º gap in the dose, corresponding to the space occupied by the C-arm fluoroscopy equipment, probably due to the absorption of scattered radiation by the equipment. There was also a drop in the intensity of the dose at the positions corresponding to the location of the head and lower members of the patient, due to the absorption of scattered radiation by the patient.

The dose rates were higher for configuration 2 than for configuration 1. That was due to the fact that the detector was in the same plane of incidence of the primary X-ray beam, meaning that there was a higher concentration of backscattered radiation^(16)^.

In this study, it was estimated that the lead surgeon receives an approximate effective dose of 34.6 µSv per procedure, which is within the range of dose values reported in the study conducted by Fuchs et al.^(14)^. Alonso et al.^(13)^ reported a dose value of 37 µSv, which is quite comparable to the value registered in the present study.

Even though the dose rate values obtained in this study are relatively low, the use of personal protective equipment is recommended^(17)^. The use of such equipment can substantially reduce radiation exposure.

During surgical interventions involving the use of radiation, most health professionals wear lead aprons and thyroid collars, although eye protection (with goggles) is rarely used.

According to the International Atomic Energy Agency, the effective dose per hip procedure received by the lead surgeon, assuming a fluoroscopy time of 25 s and the use of a 0.5-mm lead apron, should be no more than approximately 5 µSv. Considering that an X-ray beam with energy between 60 keV and 100 keV transmits 1-7% of that energy through a 0.5-mm lead apron, we can conclude that, under the conditions presented in this study and assuming that the physician is wearing a 0.5-mm lead apron, the effective dose received would be 2.5 µSv, which is below the reference value^(18)^.

On the basis of the doses estimated in this study, we can state that the use of 0.25-mm lead aprons would be sufficient to ensure safety and protection during surgical interventions involving the use of radiation. That would afford health professionals greater comfort during such procedures.

## CONCLUSION

In this study, we have shown that the radiation doses received by health professionals during fluoroscopy-guided hip surgery are low. Nevertheless, given that there are no safe levels of radiation, it is advisable to wear lead aprons, thyroid collars, and protective goggles, which can substantially reduce radiation exposure during such procedures.
